# Do open-access dermatology articles have higher citation counts than those with subscription-based access?

**DOI:** 10.1371/journal.pone.0279265

**Published:** 2022-12-22

**Authors:** Fangyi Xie, Sherief Ghozy, David F. Kallmes, Julia S. Lehman

**Affiliations:** 1 Department of Dermatology, Mayo Clinic, Rochester, Minnesota, United States of America; 2 Department of Radiology, Mayo Clinic, Rochester, Minnesota, United States of America; 3 Department of Laboratory Medicine and Pathology, Mayo Clinic, Rochester, Minnesota, United States of America; University of North Carolina at Chapel Hill Health Sciences Library: The University of North Carolina at Chapel Hill, UNITED STATES

## Abstract

**Background:**

Open-access (OA) publishing is increasingly prevalent in dermatology, and many journals now offer hybrid options, including conventional (subscription-based access [SA]) publishing or OA (with an author publishing charge) in a subscription journal. OA publishing has been noted in many disciplines, but this has been rarely studied in dermatology.

**Methods:**

Using the Clarivate Journal Citation Report, we compiled a list of English-language dermatology hybrid OA journals containing more than 5% OA articles. We sampled any OA review or original research article in 4 issues from 2018 to 2019 and matched an equal number of SA articles. Citation count, citation count excluding self-citations and view counts found using Scopus and Altmetrics score were recorded for each article. Statistical analyses were performed using logistic and negative binomial models using R software.

**Results:**

Twenty-seven hybrid dermatology journals were found, and 538 articles were sampled (269 OA, 269 SA). For both original research and review articles, OA articles had significantly higher mean citation counts (mean 13.2, standard deviation [SD] 17.0) compared to SA articles (mean 7.9, SD 8.8) (odds ratio [OR] 1.04; 95% CI 1.02–1.05; *P* < .001) including when adjusted for time from publication. Original research OA articles had significantly higher citation counts than original research SA articles (excluding self-citations; OR, 1.03; 95% CI, 1.01–1.05; *P* = .003), and review articles also had OA citation advantage than review SA articles (OR, 1.06; 95% CI, 1.02–1.11; *P* = .008). There was, however, no significant difference in citation counts between review articles and original research articles (OR, 1.00; 95% CI, 0.19–5.31; *P* = 1.000).

There was no significant difference seen in view counts (OA: mean±SD 17.7±10.8; SA: mean±SD 17.1±12.4) and Altmetric score (OA: mean±SD 13.2±47.8; SA: mean±SD 6.3±25.0) between OA and SA articles. Potential confounders included the fact that more OA articles were published in Europe than in Asia, and pharmaceutical-funded articles were more likely to be published OA.

**Conclusions:**

We noted a higher citation count for OA articles than SA articles in dermatology hybrid journals. However, dermatology researchers should take into account confounding factors when deciding whether to increase the impact of their work by selecting OA over SA publishing.

## Introduction

Open-access (OA) publishing is an increasingly popular way to disseminate scientific work, and medical researchers have the option of selecting OA for manuscript submission [1]. Traditional subscription-based journals generally rely on subscriptions to cover their publication costs (subscription-based access [SA] publishing). OA options, however, typically charge authors (or their funding sources) for accepted manuscript with article publishing charges (APCs) and are increasingly being offered by journals. Several forms of OA have developed over time. *Piwowar et al* [2] defined the different types of OA submissions. Gold OA articles are “published in an OA journal that is indexed by the Directory of Open Access Journals.” Green OA articles are published SA on the publisher page with a free copy in an OA repository. Bronze OA articles are “free to read on the publisher page, but without a clearly identifiable license.” Hybrid OA articles are free under an open license in a SA journal [2]. In this article, we also use the term ‘*hybrid journals’* to refer to journals that publish hybrid OA articles, offering either conventional SA publishing options or OA. APCs are determined by each journal separately, though APCs are typically higher in hybrid journals [3]. A recent study found 65/106 (61%) of dermatology journals were subscription journals, and 41/106 (39%) were open-access journals, although they did not specify how many of the subscription journals allowed for hybrid OA publishing [4]. They also found 4 top publishers controlled 52/106 (49%) of the publishing space in dermatology.

Researchers might be more willing to pay APCs if there was a demonstrable scientific impact benefit associated with OA submissions. Some studies have shown higher subsequent citation rates (an average number of citations received by a group of papers published in one research field in a year) with OA articles in standard journals, in some disciplines more than others [5–8]. However, this advantage is small among hybrid journals [9], and no citation advantage exists in randomized control trials [10, 11]. While randomized control trials are ideal, they can be difficult to perform without editorial input in randomizing articles to OA or SA. In this study, we examine open-access citation advantage [OACA] at the article level. Comparing articles in a hybrid journal that publishes both OA and non-OA articles has been shown to be more accurate and reduce confounders than comparing across OA and non-OA journals (examining OACA at the journal level) [9, 12]. Also, higher-impact dermatology journals tend to be hybrid journals. To our knowledge, OACA has not been studied in the field of dermatology. We hypothesize that article-level OACA exists for dermatology hybrid journals.

## Materials and methods

**Fig 1:**
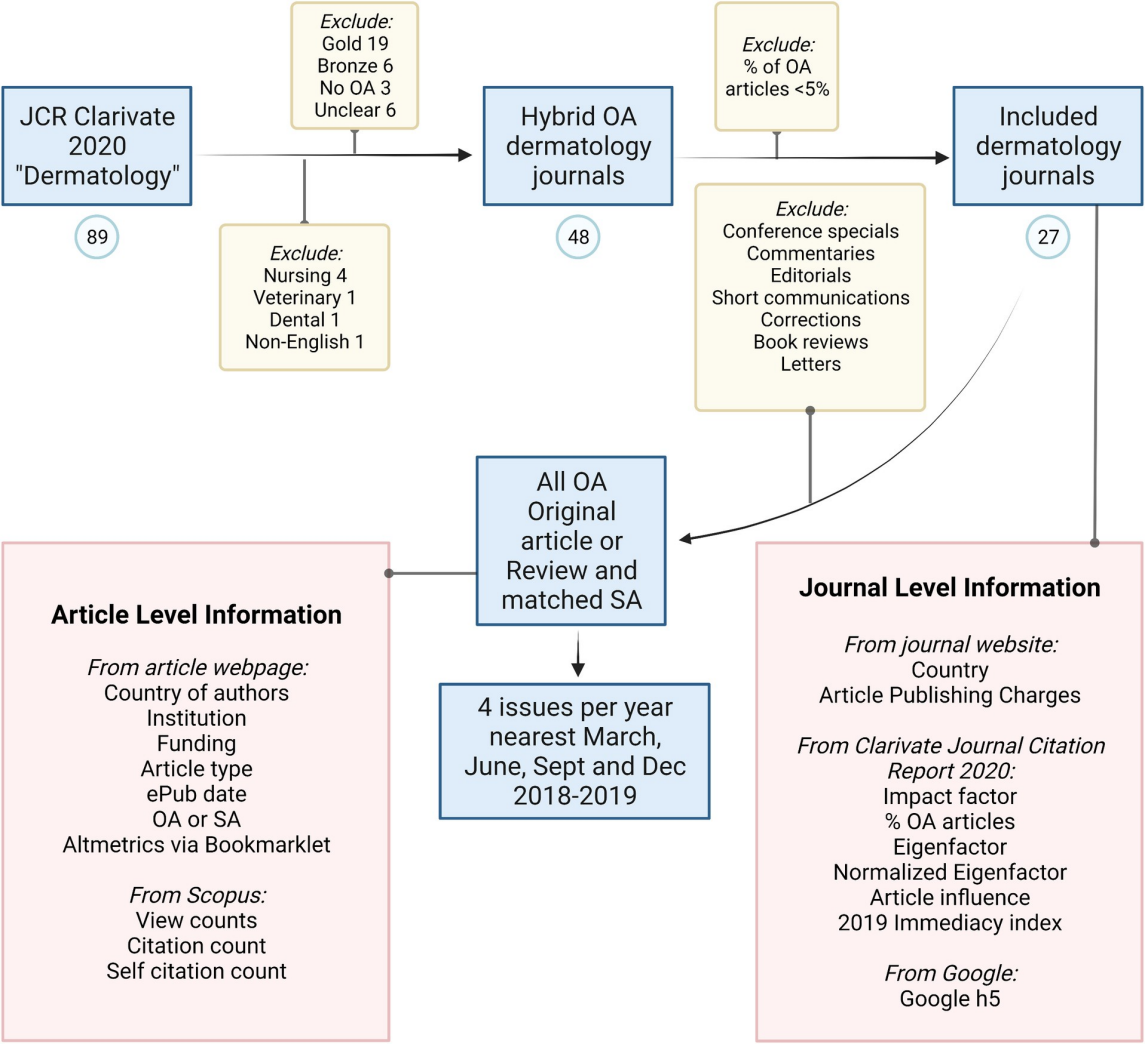
Methodology, including exclusion criteria and extracted bibliometric information, at journal and article level. OA indicates open access; SA, subscription-based access. Created with BioRender.com.

No Institutional Review Board ethical approval was required for the current study, as the data is publicly available. We compiled a list of all dermatology hybrid journals on August 6, 2021, using the Clarivate Journal Citation Report (JCR) 2020 [13], because it lists the percentage of OA articles in each journal. We included English-language journals with more than 5% OA articles, both original research and review articles. We excluded SA journals, journals containing gold or bronze OA articles, or any journal where the OA status was unclear. We also excluded hybrid journals with less than 5% OA articles due to the limited number of issues published during our sampling that would contain OA articles. Other exclusion criteria included: nursing-only, venereal, and veterinary journals (Fig 1). Within the journals sampled, we also excluded conference special issues, commentaries, editorial articles, short communications/reports, corrections, book reviews, and letters. We categorized articles into 4 groups: 1) OA original research articles, 2) SA original research articles, 3) OA review articles, and 4) SA review articles. Based on a previous study [6], we powered the study to include 38 articles in each category based on 95% CI, effect size of 0.84, α of 0.05, power of 95%, and 1:1 allocation. We selected articles published from January 1, 2018, to December 31, 2019, to reflect increasing numbers of hybrid journals while allowing time for citation accumulation and avoiding confounding COVID-19-related articles that were often automatically OA as a public health service. We sampled 4 issues each year, choosing the issues published closest to March, June, September and December for each journal to accommodate varying publishing schedules. Due to the varied number of OA articles in each issue, we selected all OA original research and review articles and a matched number of SA articles in the same category immediately adjacent to the OA articles. If there were not sufficient matching numbers of SA articles in the same issue as OA articles, we used SA articles from the following issue. If no OA articles were included in that issue, the entire issue was excluded.

All journal-level information was compiled between August 6, 2021, to August 12, 2021, (Fig 1). We extracted Journal Impact Factor (JIF), Eigenfactor, normalized Eigenfactor, Article Influence score and 2019 Immediacy Index from the Clarivate JCR 2020 when compiling the list of journals and added Google scholar h5-index (Google, Inc). Google scholar h5-index can be both article and journal-level. We included h5-index for journals listed on Google Scholar Health & Medical Sciences–Dermatology subcategory. These metrics were chosen because they are commonly available through educational institution subscription (such as ours) and commonly used journal-level metrics based on different nuanced benefits, which provide an idea of the importance of each journal to the scientific community [14, 15]. JIF is the total number of citations a journal’s articles receive over 2 years divided by the total number of published articles in those 2 years, and a higher value is considered more influential [16]. Eigenfactor score for all JCR journals adds up to 100, and higher the proportional score, the greater the impact. However as a most journals have a very small score and the normalized Eigenfactor makes interpretation easier by giving a multiple of the average score in the JCR, so anything above 1 is how many times more influential a journal is as the average journal in the JCR e.g. normalized Eigenfactor 4 is four times more influential than the average journal [17]. The Article Influence Score provides measures average influence over a 5-year period, and is based on the Eigenfactor, where a score of 5 is 5 times more influential than the average JCR journal [18]. The Immediacy Index suggests how quickly articles are cited by calculating the number of citations to articles published in one year by the number of articles published that year [19]. Google h5-index provides the h-index, e.g. h-index of 5 means the journal has 5 articles cited over 5 times, for the past 5 years [20]. We checked journal websites to confirm hybrid OA status and APCs (excluding taxes and based on original research articles no more than 6 pages in length submitted from United States). Where OA status and APCs were not available online, we contacted journals via email and excluded those that did not respond. All costs are in US$ as listed by the publishers or converted to US$ based on exchange rates on September 12, 2021. APCs were presumed to be $0 for SA articles.

We collected article-level bibliometric information (Fig 1) between August 12, 2021, and September 7, 2021, on an Excel spreadsheet. We searched the Scopus database [21] for each article citation counts, including self-citations and view counts. We collected self-citations to calculate citation counts that excluded self-citations in order to measure their influence on others. For accuracy, the country of publication, author institution, funding source, type of article, and publication date were extracted from the article page. We listed country of publication as ‘international’ if authors were from more than three countries, otherwise all countries of all participating authors were noted. We collected view counts from Scopus, which show the number of visits an article has received in the current year. The Altmetric score, a weighted count of mentions tracked by Altmetric for an individual research output, was compiled using the Altmetric Bookmarklet on the article website [22].

Data were analyzed using R software version 4.1.1 (The R Project for Statistical Computing) [23] using the Rcmdr [24] and glm2 [25] packages, with a *P* value of < .05 indicating statistical significance. Mean and standard deviation (SD) were used to represent continuous variables, while we used frequencies and percentages to represent categorical variables. We estimated the effect of OA on citation counts using a negative binomial regression model while controlling the other related predictors within the same model. The negative binomial regression model was used instead of a linear regression model because it resulted in a better fit to the data. The negative binomial regression model is similar to the Poisson regression model (for count data) except that it improves performance with data over-dispersion [26]. Moreover, we used logistic regression to identify the possible association of funding status with citation counts, Altmetrics and view counts. Logistic regression results were expressed as odds ratios (ORs) and 95% CI. Results of the negative binomial regression were expressed as incidence rate ratio (IRR) and 95% CI. Graphs were made using Prism version 8.4.2 (GraphPad for Windows, GraphPad Software, San Diego, California USA, www.graphpad.com”).

## Results

Twenty-seven journals met inclusion criteria (Table 1). We sampled 538 total articles: 232 (43%) original research articles and 37 (7%) review articles in each OA and SA group. We surpassed our original power calculation for original research articles because of the small number of review articles. Table 2 shows the breakdown of citations by sampled articles in each journal. The negative binomial regression model estimated that OA increased expected citation counts in both article types by 44.3% (IRR, 1.44; 95% CI, 1.22–1.71, *P* < .001). We examined the potential OA impact on citation counts based on article types. Similarly, review articles (OA and SA combined) and JIF increased expected citation counts by 40.9% (*P* = .02), and 14.4% (*P* < .001), respectively (Table 3). There was no significant difference in citation counts between review articles and original research articles (OR, 1.00; 95% CI, 0.19–5.31; *P* = 1.000). Funding increased the predicted citations by 12% and the presence of any US author by 18.1%, although neither of these were statistically significant. There was no significant difference seen with view counts (OA: mean±SD, 17.7±10.8; SA: mean±SD, 17.1±12.4) and Altmetric score (OA: mean±SD, 13.2±47.8; SA: mean±SD, 6.3±25.0) between OA and SA.

**Table 1:** Key bibliometric features of the 27 included dermatology hybrid journals.

Journal name	Total number of articles sampled	Country	OA cost^a^	2020 JIF	% of OA Gold	Google h5 index	Total Citations	Total Articles	Citable Items	Eigenfactor	Normalized Eigenfactor	Article Influence Score	2019 Immediacy Index
Journal of the American Academy of Dermatology	14	USA	$3,700	11.527	8.24%	93	40,257	204	263	0.003	0.652	0.753	3.191
JAMA Dermatology	10	USA	$5,000	10.282	7.41%	60	8,448	109	124	0.001	0.883	0.533	2.514
British Journal of Dermatology	64	UK	$4,200	9.302	27.20%	76	36,064	262	311	0.003	0.1	1.124	3.515
Journal of Investigative Dermatology	34	USA	$3,400	8.551	12.61%	70	37,630	209	220	0.004	0.433	0.632	2.216
American Journal of Clinical Dermatology	26	New Zealand	$4,480	7.403	18.26%	46	4,264	35	97	0.006	0.688	0.704	1.464
Contact Dermatitis	10	Denmark	$3,800	6.6	13.98%	38	7,973	112	117	0.005	1.328	0.71	2.036
Journal of the European Academy of Dermatology and Venereology	50	UK	$3,950	6.166	19.73%	NA	19,427	360	447	0.003	0.658	0.903	1.63
Journal der Deutschen Dermatologischen Gesellschaft	6	Germany	$3,300	5.584	25.39%	NA	3,597	73	119	0.006	0.125	0.734	1.154
Dermatology	4	Switzerland	$3,530	5.366	7.65%	NA	6,824	70	90	0.002	0.006	0.978	0.694
Advances in Wound Care	18	USA	$3,200	4.73	16.58%	NA	3,381	84	93	0.002	0.252	0.423	0.42
Pigment Cell & Melanoma Research	14	Denmark	$4,300	4.693	12.23%	NA	5,765	78	102	0.001	0.032	0.648	1.147
Journal of Dermatological Science	12	Ireland	$3,730	4.563	7.37%	42	6,869	73	86	0.003	0.083	0.541	0.408
Mycoses	12	Germany	$4,100	4.377	12.23%	NA	5,252	173	198	0.008	0.006	0.668	1.322
Lasers in Surgery and Medicine	16	USA	$4,100	4.025	11.86%	NA	7,332	205	212	0.007	0.616	0.576	1.086
Journal of Dermatology	18	Japan	$3,630	4.005	11.08%	38	6,895	240	261	0.003	1.719	0.464	0.551
Experimental Dermatology	10	Denmark	$4,100	3.96	12.58%	44	9,059	169	212	0.002	0.286	0.717	1.077
Wound Repair and Regeneration	20	USA	$3,300	3.617	13.79%	NA	7,277	100	112	0.002	0.155	0.623	0.471
Melanoma Research	30	USA	$3,595	3.599	6.77%	NA	3,342	79	84	0.002	0.008	0.412	0.896
Skin Pharmacology and Physiology	2	USA	$3,530	3.479	7.62%	NA	2,159	27	32	0.001	0.401	0.354	0.419
Clinical and Experimental Dermatology	4	UK	$3,100	3.47	5.49%	NA	6,342	118	166	0.002	1.56	0.475	1.254
Journal of Dermatological Treatment	24	Sweden	$3,500	3.359	8.93%	34	3,995	310	396	0.001	1.271	0.399	1.081
Photodermatology Photoimmunology & Photomedicine	12	Denmark	$3,600	3.135	10.43%	NA	1,976	53	67	0.004	0.311	0.539	0.706
Archives of Dermatological Research	16	Germany	$3,860	3.017	11.20%	NA	4,961	156	185	0.002	0.681	0.432	0.489
International Journal of Cosmetic Science	32	UK	$2,750	2.97	21.20%	NA	3,241	76	83	0.001	0.031	0.322	0.358
Journal of Cosmetic Dermatology	38	USA	$3,500	2.696	8.01%	35	3,563	651	740	0.002	1.606	0.46	0.43
Skin Research and Technology	32	Denmark	$3,800	2.365	11.23%	NA	3,035	176	180	0	6.099	0.113	0.372
Current Dermatology Reports	10	USA	$3,860	n/a	6.31%	NA	347	25	38	0.004	0.758	0.932	n/a

Abbreviations: JIF, journal impact factor; NA, not applicable; OA, open access.

^a^Article publication charge for OA excludes taxes and is based on an original research article of no more than 6 pages in length submitted from United States. All costs in US$ as listed by the publishers on September 12, 2021. Data extracted from Clarivate Journal Citation Report 2020, except for country and OA cost (extracted from journal website) and Google h5 index (from Google, Inc).

**Table 2:** Breakdown of citations in sampled articles by journal.

Journal name	Year(s) of ePublication in sampled articles^a^	Number of OA articles sampled	Number of SA articles per samples	Mean citations in sampled articles	Median citations in sampled articles	Min citations in sampled articles	Max citations in sampled articles	Total citations in sampled articles
Journal of the American Academy of Dermatology	2017–2019	7	7	9.9	8.5	0	23	138
JAMA Dermatology	2017–2019	5	5	27.0	24.5	5	61	270
British Journal of Dermatology	2016–2019	32	32	20.0	13.5	1	117	1277
Journal of Investigative Dermatology	2017–2019	17	17	17.1	12.0	1	77	583
American Journal of Clinical Dermatology	2017–2019	13	13	15.7	10.0	1	60	408
Contact Dermatitis	2018–2019	5	5	6.6	7.0	1	13	66
Journal of the European Academy of Dermatology and Venereology	2017–2019	25	25	12.9	8.5	0	85	644
Journal der Deutschen Dermatologischen Gesellschaft	2019	3	3	2.0	1.0	0	7	12
Dermatology	2018	2	2	9.8	8.5	2	20	39
Advances in Wound Care	2018–2019	9	9	10.2	3.5	0	55	184
Pigment Cell & Melanoma Research	2017–2019	7	7	8.3	7.0	1	18	116
Journal of Dermatological Science	2018–2019	6	6	4.7	2.5	0	15	56
Mycoses	2017–2019	6	6	8.6	6.5	0	22	103
Lasers in Surgery and Medicine	2017–2019	8	8	10.5	6.5	1	72	168
Journal of Dermatology	2018–2019	9	9	9.8	7.0	0	54	176
Experimental Dermatology	2018–2019	5	5	9.7	6.5	0	41	97
Wound Repair and Regeneration	2018–2019	10	10	6.6	5.0	0	22	132
Melanoma Research	2018–2019	15	15	10.1	6.5	0	41	303
Skin Pharmacology and Physiology	2019	1	1	6.0	6.0	4	8	12
Clinical and Experimental Dermatology	2017–2018	2	2	5.3	5.0	2	9	21
Journal of Dermatological Treatment	2017–2019	12	12	5.4	4.0	0	22	130
Photodermatology Photoimmunology & Photomedicine	2017–2019	6	6	4.0	2.5	0	13	48
Archives of Dermatological Research	2017–2019	8	8	4.8	4.0	0	13	77
International Journal of Cosmetic Science	2017–2019	16	16	4.3	4.0	0	12	137
Journal of Cosmetic Dermatology	2017–2019	19	19	5.3	3.5	1	26	202
Skin Research and Technology	2017–2019	16	16	4.4	3.0	0	18	140
Current Dermatology Reports	2018–2019	5	5	14.3	2.5	0	94	143

Abbreviations: OA, Open access; SA, Subscription-based access.

^a^Articles were sampled from issues in 2018 and 2019, however some articles had earlier ePublication dates.

**Table 3:** Negative binomial regression output reporting independent variable effects on citation count in Scopus.

Predictor	Incidence rate ratio (95% CI)	P value
**Open access (both article types)**	1.44 (1.22–1.71)	< .001^a^
**Funding Available**	1.12 (0.93–1.36)	.24
**Time from publication (months)**	1.03 (1.02–1.04)	< .001^a^
**Open access cost (US$)**	1.00 (1.000–1.001)	.005^a^
**View count**	1.01 (1.01–1.02)	.001^a^
**Altmetric**	1.00 (1.0001.004)	.08
**2020 JIF**	1.14 (1.10–1.19)	< .001^a^
**Review article (OA and non-OA)**	1.41 (1.06–1.88)	.02^a^
**Any author from US**	0.21 (0.01–0.42)	.04^a^

Abbreviation: JIF, journal impact factor.

^a^Statistically significant (*P* < .05)

Four articles out of 269 (1%) included in the SA group were Wiley *Free access*, meaning they were made freely accessible based on the editor’s choice; 17 of 269 (6%) were Elsevier *Open archive*, meaning freely accessible once archived and 5/269 (2%) were freely accessible after 12 months. These were not excluded from the SA group, as they only applied to specific journal policies. OACA (excluding self-citations) persisted when these 3 groups were excluded from the analysis (OR, 1.05; 95% CI, 1.03–1.07, *P* < .001).

**Fig 2:**
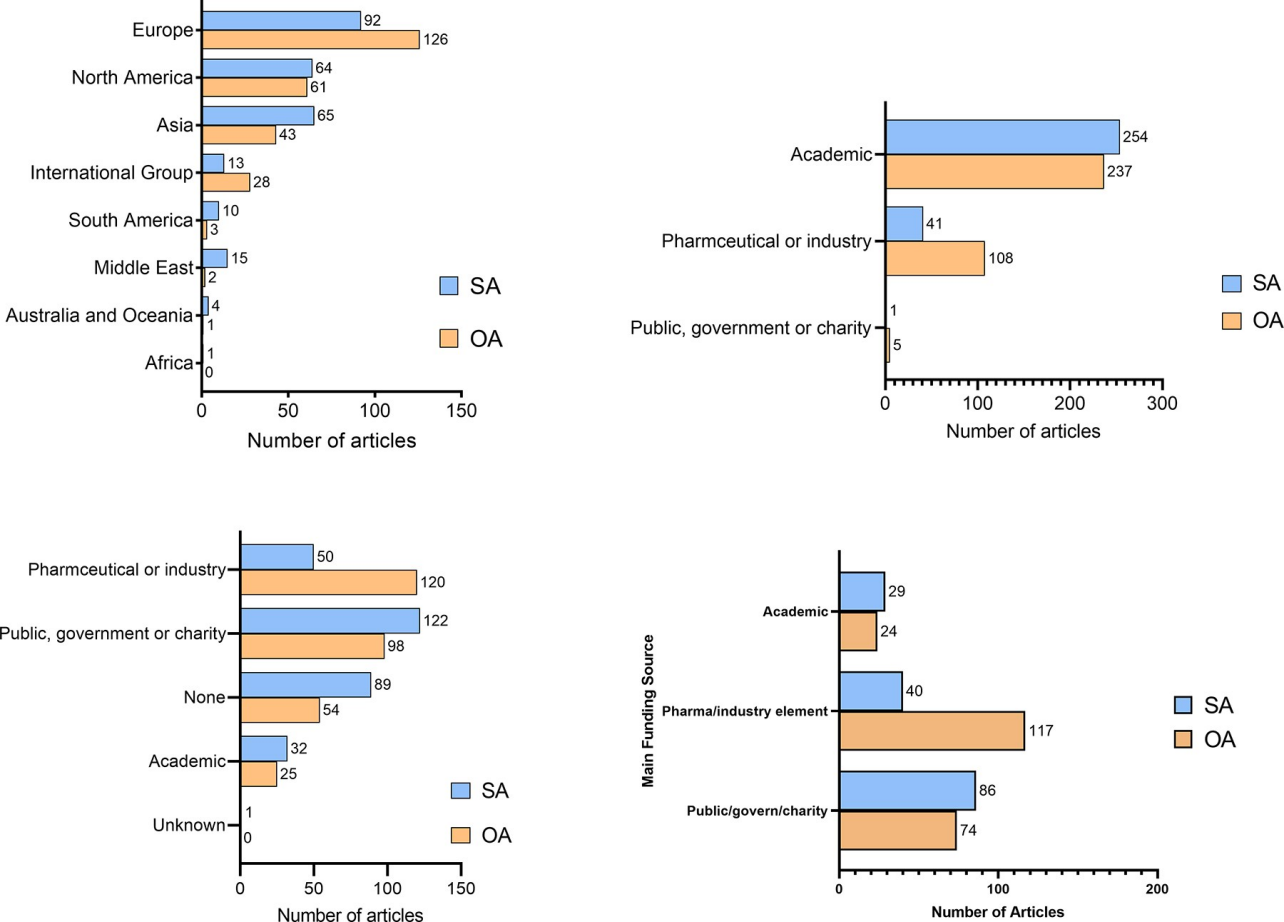
Breakdown of open access (OA) and subscription-based access (SA) articles by a) geographical region of authors, b) author institution type, c) funding source and d) article numbers by funding source.

The APCs are positively associated with the JIF (b = 0.50, 95% CI 0.42–0.57, p<0.001). Authors from Europe and authors collaborating from more than three countries (international) were more likely to publish OA articles than those from other continents, and pharmaceutical or industry authors or funders were more likely to publish OA than SA (Fig 2A–2C). There was a significantly higher rate of external funding for OA articles than SA articles (OR, 2.00; 95% CI, 1.36–2.97, *P* = .001), (Table 4). Funding had no significant effect on citation counts, Altmetric score, or view count. For studies with pharmaceutical or industry funding, more were OA (117/269, 43%) than SA (40/269, 15%) (Fig 2D).

**Table 4:** The association between funding status and different metrics.

Variables		Funding		OR (unadjusted)	OR (adjusted)^a^
		No	Yes		
**Open Access**	**No**	90 (33.5)	179 (66.5)	*Reference*	
	**Yes**	54 (20.1)	215 (79.9)	2.00 (1.36–2.97, *P* = .001^b^)	Not applicable
**Citation count in Scopus**	**Mean (SD)**	9.4 (12.8)	11.0 (14.1)	1.01 (0.99–1.03, *P* = .255)	1.01 (1.00–1.03, *P* = .123)
**Citation count in Scopus (excluding self-citations)**	**Mean (SD)**	8.1 (11.8)	8.8 (11.7)	1.01 (0.99–1.02, *P* = .513)	1.01 (0.99–1.03, *P* = .291)
**Altmetric**	**Mean (SD)**	8.0 (34.4)	10.4 (39.6)	1.00 (1.00–1.01, *P* = .514)	1.00 (1.00–1.01, *P* = .438)
**View count**	**Mean (SD)**	8.1 (11.8)	8.8 (11.7)	1.01 (0.99–1.02, *P* = .513)	1.01 (0.99–1.03, *P* = .291)

Abbreviations: OR, Odds ratio; SD, standard deviation

^a^Adjusted for time from publication to data extraction.

^b^Statistically significant.

## Discussion

We noted significantly higher citations in OA articles compared to SA articles in dermatology hybrid journals, albeit with confounders that may affect interpretation of the results. This finding may influence researchers evaluating publication options. The current study adds further OACA information in dermatology.

Some prior publications on OACA have found similar results to this current study. Within different medical specialties, prior studies have also shown OACA, with hybrid OA articles in global health cited twice as often as SA articles in the same field [27]. OACA in articles on poverty-related diseases was also reported [28]. This OACA is noted at journal level. A systematic review showed that 64 of 134 (47.8%) studies found OACA, while 37 of 134 (27.6%) found no OACA [5]. A review focused on medical journals found that OA journals had significantly higher CiteScore and percent cited (*P* < .001) than non-OA journals [7]. Articles published as hybrid OA articles are cited more than the world average, with a relative citation rate of 1.31 [2]. One study comparing OA and SA articles in a single hybrid journal found increased view counts and downloads in OA articles that was sustained over time [29].

Some studies, however, found contrasting results to the current study. One author showed a significant OACA at the article level in only 2 of 11 journals, and overall a small (17%) increase in citations of OA articles compared to SA articles, which weakened over time [9]. This advantage was noted more for medical and health sciences than for agricultural sciences, which may explain why 9 of 11 journals had no OACA while our study focused purely on dermatology journals. More robust randomized control trials suggest the main advantage of OA appears to be more initial browsing than incorporation into future work [10, 11]. Davis *et al* [11] showed that OA articles had more full text and PDF downloads than SA articles in the first 6 months following publication, but no OACA at 1 year or at 3 years following publication [10]. The differing result may be explained by the longer follow-up and randomization, which removes potential confounders such as author origin and funding. A systematic review of radiology journals found no statistical difference in citations between OA and non-OA journals [30]; however, this review looked at OACA at the journal level not at the article level, as in the current study.

The view count and Altmetric score were not significantly different between OA and SA articles in the current study, possibly reflecting that view counts measure views of the title and abstract page of an article but not necessarily the full article [31]. Informal ways of browsing such as physical library journals or a colleague emailing a PDF of interest may be difficult to measure with bibliometrics [10]. In another study, mean Altmetric scores have been shown to be significantly higher in OA than SA articles in intensive care medicine (17.34 vs 8.45, *P* < .01) though not at 1-year follow-up, and not in anesthesia [32]. However, the same study did show that Altmetric score was associated with citation count 18 month following publication. Some authors argue that researchers may publish their higher quality work as OA, leading to more citations for OA articles due to the increased quality of work rather than publication option [33]. However, another study noted that advantage is independent of author self-selection [34].

We found that articles with authors from Europe and North America were more likely to be OA, as were pharmaceutical- or industry-funded articles, perhaps due to the substantial cost of APCs with OA publishing. We hypothesize that this finding may have been due to the substantial cost of APCs with OA publishing, which may be more affordable to industry sponsors or investigators in high-income countries and reflects a prior study that shows North American and European researchers are the predominant authors of OA articles [6]. Institutions in Europe were more likely to publish OA articles, perhaps due in part to more European institutions having prepaid APCs for their authors [35]. Many publishers do offer reduced or waived APCs for low- or middle-income countries, however we did not eliminate this confounder when we designed the study. These are undoubtedly confounders that may affect interpretation of our results (e.g. pharmaceutical-funded studies may be cited more than their counterparts).

Previous research found no correlation between JIF and APCs at the journal level in gold OA journals [15]. However, we found a positive correlation between citations and cost to publish in the current study, which is likely confounded by the APCs associated with OA articles. Even within OA articles, APCs vary, and a higher impact journal may charge more for publication as they have larger editorial operations and need to cover the cost of more rejected articles [3].

A prior review highlighted the high risk of bias in OACA articles in population, data collection, study design, and results [5]. While we tried to control for some bias, limitations and biases that exist in our study include unavoidable confounders, such as article topic and author prestige. We did not study articles published in 2020 and 2021 because COVID-19 articles are often published as OA, so more recent trends may be missed. Also, COVID-19 may have affected citation counts of articles published in 2019 and be a potential source of bias. We did not examine all article types, and sampled articles across 2 years; therefore, the noted OACA may not be generalizable to all publication venues. We studied hybrid journals to reduce the confounding factors of journals publishing OA or SA articles exclusively, but there may be differences in citations for purely OA journals compared to hybrid journals. There are more advanced article-level metrics, including field-weighted citation impact and citation benchmark in Scopus, which are time- and field- normalized. We did not collect this information and opted for more commonly used citation count and counts excluding self-citations. We suggest that future studies incorporate these article-level metrics. APCs studied do not take into account waivers or reduced fees for institutions or low-income countries. Citation counts and Altmetric scores are only a rough proxy for the actual scientific impact of an article, and perhaps scientific impact in dermatology is better measured by translation into clinical practice e.g. the direct impact of adoption of a drug tested and recommended in a clinical trial [36].

## Conclusions

OACA is observed in dermatology hybrid journals. However, results should be interpreted bearing in mind confounders when determining whether researchers increase the impact of their work by selecting OA over SA publishing.

## Supporting information

S1 File Data files. (XLSX)

## References

[1] Laakso M, Welling P, Bukvova H, Nyman L, Bjork BC, Hedlund T. The development of open access journal publishing from 1993 to 2009. PLOS One. 2011;6(6):e20961. Epub 2011/06/23. doi: 10.1371/journal.pone.0020961 ; PubMed Central PMCID: PMC3113847. 21695139 PMC3113847

[2] Piwowar H, Priem J, Lariviere V, Alperin JP, Matthias L, Norlander B, et al. The state of OA: a large-scale analysis of the prevalence and impact of Open Access articles. PeerJ. 2018;6:e4375. Epub 2018/02/20. doi: 10.7717/peerj.4375 ; PubMed Central PMCID: PMC5815332. 29456894 PMC5815332

[3] Van Noorden R. Open access: The true cost of science publishing. Nature. 2013;495(7442):426–9. Epub 2013/03/30. doi: 10.1038/495426a . 23538808

[4] Ding J, Vijayasarathi A, Amornteerasawas OM, Hiebert G, Khosa F. Subscription-based and open access dermatology journals: the publication model dilemma. Dermatol Online J. 2022;28(2). Epub 2022/06/08. doi: 10.5070/D328257392 . 35670679

[5] Langham-Putrow A, Bakker C, Riegelman A. Is the open access citation advantage real? A systematic review of the citation of open access and subscription-based articles. PLOS One. 2021;16(6):e0253129. Epub 2021/06/24. doi: 10.1371/journal.pone.0253129 ; PubMed Central PMCID: PMC8221498. 34161369 PMC8221498

[6] Norris M, Oppenheim C, Rowland F. The citation advantage of open-access articles. J Am Soc Inf Sci Tec. 2008;59(12):1963–72. doi: 10.1002/asi.20898 WOS:000259455500008.

[7] AlRyalat SA, Saleh M, Alaqraa M, Alfukaha A, Alkayed Y, Abaza M, et al. The impact of the open-access status on journal indices: a review of medical journals. F1000Res. 2019;8:266. Epub 2019/04/20. doi: 10.12688/f1000research.17979.1 ; PubMed Central PMCID: PMC6449789. 31001420 PMC6449789

[8] ClarivateAnalytics. InCites Essential Science Indicators Help [7/25/2022]. Available from: http://help.incites.clarivate.com/incitesLiveESI/ESIGroup/indicatorsGroup/fieldBaselines/citationRatesBaselines.html#:~:text=A%20citation%20rate%20is%20the,of%20papers%20in%20the%20group.

[9] Davis PM. Author-Choice Open-Access Publishing in the Biological and Medical Literature: A Citation Analysis. J Am Soc Inf Sci Tec. 2009;60(1):3–8. doi: 10.1002/asi.20965 WOS:000262424900002.

[10] Davis PM. Open access, readership, citations: a randomized controlled trial of scientific journal publishing. FASEB J. 2011;25(7):2129–34. Epub 2011/04/01. doi: 10.1096/fj.11-183988 . 21450907

[11] Davis PM, Lewenstein BV, Simon DH, Booth JG, Connolly MJ. Open access publishing, article downloads, and citations: randomised controlled trial. BMJ. 2008;337:a568. Epub 2008/08/02. doi: 10.1136/bmj.a568 ; PubMed Central PMCID: PMC2492576. 18669565 PMC2492576

[12] Harnad S, Brody T. Comparing the Impact of Open Access (OA) vs. Non-OA Articles in the Same Journals. D-Lib Magazine. 2004;10(6):1–.

[13] Clarivate. Journal Citation Reports 2021 [01/09/2021]. Available from: https://jcr.clarivate.com/jcr/home.

[14] Yuen J. Comparison of Impact Factor, Eigenfactor Metrics, and SCImago Journal Rank Indicator and h-index for Neurosurgical and Spinal Surgical Journals. World Neurosurg. 2018;119:e328–e37. Epub 2018/07/29. doi: 10.1016/j.wneu.2018.07.144 . 30055360

[15] Yuen J, Muquit S, Whitfield PC. Correlation Between Cost of Publication and Journal Impact. Comprehensive Cross-sectional Study of Exclusively Open-Access Surgical Journals. J Surg Educ. 2019;76(1):107–19. Epub 2018/08/14. doi: 10.1016/j.jsurg.2018.06.029 . 30100322

[16] Garfield E. The Clarivate Analytics Impact Factor: Clarivate Analytics; 1994 [07/27/2022]. Available from: https://clarivate.com/webofsciencegroup/essays/impact-factor/#:~:text=The%20annual%20JCR%20impact%20factor,years%20(see%20Figure%201).

[17] West J. A Closer Look at the Eigenfactor™ Metrics Clarivate2017 [3/18/21]. Available from: https://clarivate.com/blog/closer-look-eigenfactor-metrics/#:~:text=To%20improve%20the%20interpretation%20and,of%20journals%20in%20the%20JCR.

[18] Clarivate_JCR_Help. Article Influence Score 2021 [07/27/2022]. Available from: https://jcr.help.clarivate.com/Content/glossary-article-influence-score.htm#:~:text=The%20Article%20Influence%20Score%20determines,all%20articles%20in%20all%20publications.

[19] Clarivate_JCR_Help. Immediacy Index 2021 [07/27/2022]. Available from: https://help.incites.clarivate.com/incitesLiveJCR/glossaryAZgroup/g7/7751-TRS.html.

[20] Scholar G. Google Scholar Metrics 2022 [07/27/2022]. Available from: https://scholar.google.com/intl/en/scholar/metrics.html#metrics.

[21] Elsevier. Scopus 2021 [05/09/2021]. Available from: https://www.scopus.com/search/form.uri?display=authorLookup#basic.

[22] Altmetric. Altmetric for Researchers 2021 [01/09/2021]. Available from: https://www.altmetric.com/audience/researchers/.

[23] Team RC. R: A Language and Environment for Statistical Computing. Vienna, Austria: R Foundation for Statistical Computing; 2017.

[24] Fox J, Bouchet-Valat M, Andronic L, Ash M, Boye T, Calza S, et al. Rcmdr: R Commander. 2.6–2 ed2020.

[25] package IM, Donoghoe MW. glm2: Fitting Generalized Linear Models. 1.2.1 ed2018.

[26] Hilbe JM. Negative binomial regression: Cambridge University Press; 2011.

[27] Smith E, Haustein S, Mongeon P, Shu F, Ridde V, Lariviere V. Knowledge sharing in global health research—the impact, uptake and cost of open access to scholarly literature. Health Res Policy Syst. 2017;15(1):73. Epub 2017/08/31. doi: 10.1186/s12961-017-0235-3 ; PubMed Central PMCID: PMC5576373. 28851401 PMC5576373

[28] Breugelmans JG, Roberge G, Tippett C, Durning M, Struck DB, Makanga MM. Scientific impact increases when researchers publish in open access and international collaboration: A bibliometric analysis on poverty-related disease papers. PLoS One. 2018;13(9):e0203156. Epub 2018/09/20. doi: 10.1371/journal.pone.0203156 . 30231044 PMC6145557

[29] Wang XW, Liu C, Mao WL, Fang ZC. The open access advantage considering citation, article usage and social media attention (vol 103, pg 555, 2015). Scientometrics. 2015;103(3):1149–. doi: 10.1007/s11192-015-1589-3 WOS:000354489600019.

[30] Narayan A, Lobner K, Fritz J. Open Access Journal Policies: A Systematic Analysis of Radiology Journals. J Am Coll Radiol. 2018;15(2):237–42. Epub 2017/12/16. doi: 10.1016/j.jacr.2017.10.012 . 29242023

[31] Floyd AR, Wiley ZC, Boyd CJ, Roth CG. Examining the Relationship between Altmetric Score and Traditional Bibliometrics in the Pathology Literature. J Pathol Inform. 2021;12:8–. doi: 10.4103/jpi.jpi_81_20 . 34012712 PMC8112340

[32] Black CS, Lehane DJ, Burns C, O’Donnell BD. An examination of the effect of open versus paywalled access publication on the disseminative impact and citation count of publications in intensive care medicine and anesthesia. J Crit Care. 2018;46:88–93. Epub 2018/05/29. doi: 10.1016/j.jcrc.2018.05.008 . 29804038

[33] Craig ID PA, McVeigh ME, Pringle J, Amin M. Do Open Access Articles Have Greater Citation Impact? A critical review of the literature. Publishing Research Consortium, J Informetr. 2007;1(3):239–48.

[34] Gargouri Y, Hajjem C, Lariviere V, Gingras Y, Carr L, Brody T, et al. Self-selected or mandated, open access increases citation impact for higher quality research. PLOS One. 2010;5(10):e13636. Epub 2010/10/27. doi: 10.1371/journal.pone.0013636 ; PubMed Central PMCID: PMC2956678. 20976155 PMC2956678

[35] Wiley. Open Access Funds–Payments Made Easy 2021 [13/09/2021]. Available from: https://authorservices.wiley.com/author-resources/Journal-Authors/open-access/affiliation-policies-payments/institutional-funder-payments.html.

[36] Williams HC, Rogers NK, Chalmers JR, Thomas KS. Scoping the international impact from four independent national dermatology trials. Clin Exp Dermatol. 2021;46(4):657–62. Epub 2020/11/13. doi: 10.1111/ced.14506 . 33179251

